# Do Natural Disasters Affect Voting Behavior? Evidence from Croatian Floods

**DOI:** 10.1371/currents.dis.cbf57c8ac3b239ba51ccc801d3362c07

**Published:** 2018-04-06

**Authors:** Kosta Bovan, Benjamin Banai, Irena Pavela Banai

**Affiliations:** University of Zagreb; University of Zadar; University of Zadar

## Abstract

**Introduction:**

Studies show that natural disasters influence voters’ perception of incumbent politicians. To investigate whether voters are prone to punish politicians for events that are out of their control, this study was conducted in the previously unstudied context of Croatia, and by considering some of the methodological issues of previous studies.

**Method:**

Matching method technique was used, which ensures that affected and non-affected areas are matched on several control variables. The cases of natural disaster in the present study were floods that affected Croatia in 2014 and 2015.

**Results:**

Main results showed that, prior to matching, floods had an impact on voting behaviour in the 2014 and 2015 elections. Voters from flooded areas decreased their support for the incumbent government and president in the elections following the floods. However, once we accounted for differences in control variables between flooded and non-flooded areas, the flood effect disappeared. Furthermore, results showed that neither the presence nor the amount of the government’s relief spending had an impact on voting behaviour. Discussion: Presented results imply that floods did not have an impact on the election outcome. Results are interpreted in light of the retrospective voter model.

## Introduction

Some of the ongoing questions regarding voting behaviour are related to voters’ competencies. Can citizens correctly perceive information or are they prone to biases ^1,2^ are they informed enough ^3,4^ ^5^ are they motivated to participate in politics 6 , etc.? These questions relate to the idea, or the norm, of a rational citizenry. They are of great importance because assumptions and findings on voters’ rationality are used to justify different political institutions and systems 7. If voters are rational, their broad political participation and inclusion should be warranted; if they are not, their role in the political system should be minimised.

**(Ir)rational voters**

One of the main models of rational voting is the retrospective voter. This model is based on the idea that citizens use voting to punish or reward incumbents 8. In that sense, elections are used to control the democratic processes and hold politicians accountable for their actions 9. Politicians, in turn, are incentivised to keep their promises and to perform well during their mandate. The evaluation, or ‘performing well’, is usually conceptualised via economic indicators, and connected with the concept of economic voting^10^^,^^11^^,^^12^. Studies mostly use either macro-level indicators (sociotropic voting), such as inflation, GDP, economic growth, etc. or micro-level indicators, such as individual changes in economic welfare (egotropic, pocketbook voting)^10^^,^^13^.

If the economy, either on a national or individual level, is doing well, voters will reward the incumbent by voting for her/him in the upcoming election. While some researchers claim that the retrospective economic voting is a robust phenomenon 11^,^^14^, other authors point out two caveats.First, it seems that economic voting depends on contextual factors, such as the number of political alternatives 10, media attention 15, or incumbents' ideology 16. Second, and more important for the idea of retrospective voting, are findings that show that citizens do not know enough about politics, are prone to biases, and hold the government accountable for things which are out of its control 17.

The first set of objections to retrospective voting does not necessarily endanger the concept of voters’ rationality. Citizens can still be prospectively rational, i.e. vote for the party or candidate whose positions on political issues are closest to their positions, known in literature as issue voting 18^,^^19^^,^^20^ .

On the contrary, the other set seriously undermines the concept of voters’ rationality. For example, how can citizens evaluate government success if most of them do not know what parties form the government 12? Furthermore, it is not just that citizens do not know enough about politics; studies have shown that citizens base their evaluations of candidates on politically irrelevant characteristics, like the candidate’s physical appearance or vocal characteristics 21^,^^22^^,^^23^^,^^24^^,^^25^^,^^26^ . It is assumed that these physical characteristics are related to candidates’ leadership skills. More masculine facial and vocal features signal dominant behaviour and good leadership qualities, and those findings hold in cross-cultural contexts and in different political systems. Besides candidates’ traits, some external factors, which are beyond the control of politicians, could affect voters’ decisions. One example of an external factor are natural disasters.

**Natural disasters and voting behavior**

It has been shown that natural disasters, as well as some other negative events, can influence voters’ perception of incumbent politicians and the government. Perhaps the most striking results are reported by Achen and Bartels 27^,^^28^. They showed that shark attacks in New Jersey in 1916 decreased President Wilson’s vote share in the election held the same year. Authors also examined the effects of droughts and floods on voting behaviour in the 1896-2000 period and reported that floods had a negative effect on electoral support for the incumbent president’s party. Moreover, they point out that these irrationalities were the result of egotropic retrospective voting, by which citizens could not correctly attribute the changes in their economic status. The main assumption is that citizens engaged in blind retrospection, which means that when voters “are in pain they are likely to kick the government… In most cases, incumbents will pay at the polls for bad times, whether or not objective observers can find a rational basis for blame” 28 (p.118). This has implications for accountability in democracy, and Achen and Bartels 29 (p. 23) conclude that it “significantly degrades the efficacy of elections as mechanisms for inducing incumbent leaders to pursue their citizens’ subjective well-being”.

Since then, several studies have focused on the effects of irrelevant random events on voting behaviour. For example, it has been found that a win by the local college football team before Election Day led to an increase in the vote share of the incumbent party in presidential, senatorial, and gubernatorial elections in the team’s home county 30. Moreover, it has been shown that voters punished the incumbent party in presidential elections for damage caused by tornadoes 31. Furthermore, Gasper and Revees 32 reported that voters “punish” both presidents and governors for weather events. In a similar vein, using data on Indian elections, it has been reported that rainfall, just one standard deviation from the optimal level, reduced the vote share for incumbent coalitions by 3.25 percentage points 33. In addition, different types of natural disasters, like earthquakes, landslides, volcanic eruptions, and floods increase the likelihood of a political crisis through anti-government demonstrations, riots, guerrilla warfare, and intra-state conflict 34. Chang and Berdiev 35, using data from 156 countries in a period of 35 years, found that natural disasters, like floods, extreme temperatures, earthquakes, and windstorms, increased the probability that an incumbent government is replaced. Natural disasters have an impact on turnout as well. Using data on Hurricane Katrina, Sinclair, Hall and Alvarez 36 showed that flooding decreased participation in the following election. However, authors also showed that voters who experienced more than 6 ft of flooding were more likely to vote, compared to those who experienced less flooding. Furthermore, after the 2010-2011 floods in Pakistan, turnout was higher among citizens from impacted areas 37 (but see 38).

As pointed out above, there is an extensive body of research that shows that citizens punish incumbents at elections following a natural disaster, which might seem irrational. The reason for this could be that natural disasters often cause resource scarcity, which leads to unequal distribution of resources, and may lead to low satisfaction with the present government, although it did not cause the scarcity 35. At first glance, it seems that these findings put democratic accountability in a tough position. However, Healy and Malhotra 17 point out that voters can and should hold government accountable for failure in relief after disasters. From their perspective, it could be even rational for voters to respond negatively to disasters because any negative event could be a signal of the incumbent’s competencies. Since it is costly to distinguish if that was the case, it is logical to punish the government, as a sort of “just-in-case” scenario. Moreover, the government could be responsible with respect to the way it deals with the disaster. For example, Arceneaux and Stein 39 investigated the effects of massive flooding after Tropical Storm Allison in Houston, which happened a few months before the mayoral election in 2001. They have found that, even though the government could not be blamed for natural disasters, voters evaluated government performance in terms of how it handled the disaster (see also Lay 40). If voters believed that the government could have done more to prevent the level of damage, they were willing to attribute blame and punish incumbents accordingly. Similar results were found in a study of effects of tornado damage on voting behavior 31. The incumbent government lost voters’ support when a formal declaration of natural disaster was absent. This finding suggests that voters punish the incumbent party if they perceive lack of competence in dealing with natural events. However, voters also reward incumbents. Healy and Malhotra 41 showed that relief spending had a positive impact on the vote, and Velez and Martin^42^ found that Hurricane Sandy had a positive impact on re-electing Barack Obama. Overall, it seems that voters’ immediate response to natural disasters is to punish incumbents, but if they perform well, i.e. give out disaster declarations or approve relief spending, they can counteract the negative tendency 32.

In order to investigate whether voters are indeed prone to punish politicians for events that are out of their control, the present study focuses on a previous unstudied context - the case of 2014’s and 2015’s floods in Croatia. Furthermore, we take into account some of the methodological issues of previous studies. The starting point was a recent study conducted by Fowler and Hall 43. They criticised Achen and Bartel’s 27 original study for the way certain counties were included/excluded, pointing out that 1916 shark attacks were not in any way a peculiar case of shark attacks. Fowler and Hall 43 collected data on every fatal shark attack in U.S. history and county-level returns from every presidential election in the period between 1872 and 2012 and found no effect of shark attacks on the president’s vote share. Based on their finding, the authors concluded that Achen and Bartel’s 27 results were most likely a false positive (for a similar re-evaluation of football game effects on voting behaviour see Fowler & Montagnes 44). Keeping these contradictory findings in mind, we conceptualised natural disasters as a form of natural experiment with an experimental and control group. We were also aware that disasters do not create equivalent groups, which means that we cannot just assume that our research has the form of a natural experiment, we need to ensure that this indeed is the case 38. One way to achieve this is by using a matching method technique. This technique ensures that all areas affected by natural events are matched to non-affected areas on several control variables which are important for explaining the vote share (e.g. lagged vote share, unemployment rate, mean income, population size, etc.). In this way, “starting” differences between affected and non-affected counties are accounted for, which leads to more reliable results regarding the impact of natural disasters on voting behaviour (for an example see 42).

**Floods in Croatia**

During 2014, a total of 148 Croatian municipalities experienced floods, with two major events being floods in February and May. During the February floods, the Sisak and Karlovac areas were affected by high water levels in the rivers Sava, Kupa, and Odra. For ten days, in certain areas, the Sava had water levels over 7.7 metres, the Kupa had a maximum water level of 8.27 metres, and water levels at the Odra mouth were 8.9 metres, which was a historical maximum 45. Due to above average rainfall in April 2014, the Sava with its tributaries experienced extreme water levels during the May floods. The most affected were Vukovar-Srijem and Brod-Posavina Counties, for which 214 kilometres of Sava were defended against overflowing. For all eight measuring stations in that area, water levels were higher than the historical maximum (sometimes over a metre higher). At certain places the embankments did not hold, causing floods in municipalities (such as Rajevo Selo, Gunja, and Račinovci), and evacuations of residents 46. TThe government response was swift with almost four thousand firefighters and soldiers deployed. Among other things, over 1.5 million bags of sand and over 300 hundred machines were stationed in the affected regions 47. To help the affected areas, the public raised over 50 million HRK (≈7,400,000 USD)^48^. The governmental officials, including the Prime Minister, Minister of Internal Affairs, Minister of Agriculture, and the President of Croatia, were present in the affected areas. However, there was public outrage at the way the floods were handled, mainly because the embankments did not hold the water 49.

Compared to the floods in 2014, the number of floods was much lower in 2015, with 34 municipalities being affected. The floods in May and October were less severe than those from the previous year, with the Samobor and Karlovac areas being hit. Still, at certain areas, the embankments did not hold, and the water level of the Kupa was higher than 8 metres. Due to better weather conditions, and more work being done for the reconstruction and improvement of the embankments, 2015 was characterised as being a “normal” year for floods 50.

In Croatia, activities regarding floods are bound by the legal framework on natural disasters. The Croatian Act on Protection against Natural Disasters 70 states that the mayor of a county oversees the declaring of a natural disaster (art. 6). After a natural disaster is declared, a county’s Committee for Natural Disasters, following the prescribed Methodology for making threat assessments 71, assesses the damage for all municipalities within the county (art. 38, par. 2). The assessments are reviewed by the National Committee for Natural Disasters, which proposes the amount of relief spending (art. 38, par. 1). The final approval of relief spending is made by the government (art. 33, par. 3).

**The present study**

The goal of this study was twofold. First, we wanted to investigate whether floods had an impact on the following elections, i.e. did Croatian citizens in flooded areas punish incumbents for the floods. Second, we wanted to examine whether relief spending had an impact on the voting behaviour of citizens affected by the floods. In other words, did citizens in flooded areas reward or punish incumbents based on the presence of financial help? To answer these research questions, a natural experiment was conducted, with independent variables being presence of flood and presence of relief spending, and dependent variables being change in incumbent’s vote share and turnout from previous to the current election.

Data from four elections was used: the 2009 and 2014 presidential elections, and the 2011 and 2015 parliamentary elections. Both presidential and parliamentary elections featured an incumbent running for re-election. For each incumbent, we used his (or his party's) electoral success from the previous election (i.e. success in the 2014 presidential election was compared with success in the 2009 presidential election, and for the 2015 parliamentary election, we used data on electoral success in the 2011 parliamentary election). This way we could take into account the overall change in support within each election cycle. The first round of the presidential election was held on 28th December 2014, so we were able to investigate more immediate effects of the 2014 floods on voting behaviour. To investigate more long-term effects of floods, we used the parliamentary election which was held on 8th November 2015. This election was also used to see if relief spending had an impact on voting behaviour.

## Method

**Research design**

In the present study, floods were considered as a natural experiment, where some municipalities formed a treatment group (flooded areas) and others a control group (non-flooded areas). This should not imply that causal mechanisms can be easily discussed because one cannot be a priori certain that the only difference between these two groups is exposure to floods. Lay 40 (p. 649) points this out by stating that “disasters do not affect all people equally”. Additionally, this method falls within the scope of observational design in which “treatment is not randomly assigned but rather passively observed” 51. This means that the differences in dependent variables can be a result of bias, and not the treatment itself. Since floods cannot be controlled as an experimental variable, and random assignment of municipalities to either group is not possible, we are forced to deal with this issue post hoc. While this can be done via regression models, there are several issues with that type of analysis that can result in remaining biases that confound the causal findings (more details in 52^,^^53^). To address this issue, researchers have focused on the use of propensity scores, which are a “probability…that a subject will be treated based on an observed group of covariates.”^52^ (p. 954; emphasis added). ). Propensity scores are used in a wide array of research areas 54 and have recently been used in assessing the impact of natural disasters on voting 42^,^^55^. By using propensity scores, we are able to calculate a unified probability of being affected by floods for each municipality, based on several variables that could impact the dependent variable (voting decision).

Next, based on the propensity scores, it was possible to create a subsample of municipalities that were not affected by floods and match them to those municipalities that were affected. In the present study, the nearest neighbour matching algorithm was employed 54 . Matches were formed based on the nearest propensity score. This method was adequate for our sample because the subsample of control (non-flooded) municipalities was large enough (N_presidential election_ = 404, N_parliamentary election_ = 402)^56^. To form propensity scores, several variables related to voting behaviour were used: unemployment rates, income per capita, municipal income per capita, level of education, lagged incumbent vote share, and lagged turnout.

**Data collection**

Three sets of data were collected: a) data on election results, b) data on flooded areas, and c) information used to form propensity scores.

a) *Data on election results*. Relevant information about the presidential and parliamentary elections was obtained from the State Electoral Commission of the Republic of Croatia (http://www.izbori.hr/). For each election, data was obtained in sheet form for each electoral district and was later combined into one sheet with data for each municipality. Municipality level data on the number of voters, total votes, and votes for the current incumbent was collected for elections before the floods (2009 and 2011) and elections following the floods (2014 and 2015).

b) *Data on flooded areas*. To identify flooded municipalities, we contacted each county (total of 21 counties, including the capital Zagreb as its own county), and received a formal assessment for which of the municipalities a natural disaster, specifically a flood, was proclaimed in 2014 and 2015. In addition, information on damage appraisal was obtained. To obtain data on relief spending, governmental decisions, which are available online (http://www.mfin.hr/hr/odluke-vlade-rh-o-dodjeli-sredstava-pomoci), were collected for all flood related relief spending in the period 2014-2015 for each municipality.

c) *Information used to form propensity scores*. In order to form propensity scores, to produce samples similar in their demographics, data on unemployment rates, income per capita, municipal income per capita, and level of education were collected from the Ministry of Regional Development and European Union funds (https://razvoj.gov.hr/o-ministarstvu/regionalnirazvoj/indeks-razvijenosti/112). The Ministry periodically measures these variables to form a development index (a composite measure of socioeconomic variables). This data was downloaded from the Ministry’s website for each municipality in Croatia. We used data from 2013, which was the last data point available.

## Results

Data was analysed with R 3.2.4., using MatchIt 57, dplyr 58, lm.beta 59, and car 60 packages. Analyses were done separately for presidential and parliamentary elections. Independent variables were the presence of flood and presence of relief spending, and dependent variables were change in incumbent’s vote share and turnout in the current election compared to previous.

For each of the elections, we reported results obtained before and after matching the affected and non-affected areas in key variables: unemployment rate, income per capita, municipal income per capita, level of education, lagged incumbent vote share, and lagged turnout. In addition, we investigated the impact of relief spending on the 2015 parliamentary election.

**Flood impact on 2014 presidential election**

To investigate the flood effect on the 2014 presidential election held in December, data on floods that took place in 2014 during February and May was used. Main results regarding flood effect on voting behaviour in the 2014 presidential election are reported in Table 1. Before matching flooded and non-flooded areas, results showed a significant difference between flooded and non-flooded municipalities in the decrease in support for the incumbent president. Flooded areas showed a higher drop in incumbent vote share in the election following the floods. At first glance, it might seem that voters in flooded areas punished the incumbent. However, there were also significant differences in all socioeconomic variables between flooded and non-flooded areas. Unflooded areas had lower unemployment, higher individual and municipal income per capita, and higher education level. This implied that the matching of affected and non-affected municipalities was warranted. To match the subsamples, MatchIt R package was used and the nearest neighbour method was applied. This resulted in a new sub-sample of non-flooded municipalities (the rest were excluded from the analysis).

After matching, there was no significant difference in any of the socioeconomic factors. Analysis using matched samples also revealed that there was no difference in turnout, nor in the incumbent vote. This means that, once we accounted for relevant variables, the difference between flooded and non-flooded areas in vote change disappeared. In other words, the impact of floods on the 2014 presidential election was absent.

**Table 1:** Table 1. Flood effects on 2014 presidential election for matched and unmatched samples

	Unmatched data		Matched data	
	Non-flooded	Flooded	Non-flooded	Flooded
Unemployment	17.34 (8.78)	23.46*** (9.1)	22.98 (9.67)	23.46 (9.1)
Income	22471.44 (6119.15)	19237.45*** (5288.13)	19299.04 (5620.64)	19237.45 (5288.13)
Municipal income	2220.85 (1830.85)	1340.43*** (1033.65)	1196.84 (824.99)	1340.43 (1033.65)
Education	70.56 (10.91)	63.23*** (9.74)	63.63 (10.53)	63.23 (9.74)
Lagged turnout (2009)	49.05 (8.94)	49.29 (7.51)	48.69 (10.03)	49.29 (7.51)
Lagged vote (2009)	60.64 (17.11)	60.64 (17.11)	61.39 (16.44)	60.09 (11.37)
Turnout change (2014 - 2009)	8.20 (6.64)	7.89 (5.4)	8.70 (6.38)	7.89 (5.40)
Vote change (2014 - 2009)	-13.12 (7.56)	-14.82* (6.62)	-13.37 (8.64)	-14.82 (6.62)
N	404	148	148	148

Sample means with standard deviation in the parenthesis for: Unemployment – average percentage of unemployed individuals 2010-2012, Income – average yearly income per capita 2010-2012 (in HRK), Municipal income – average yearly municipal income per capita 2010-2012 (in HRK), Education – percentage of population aged 16-65 with high school of higher 2011, Lagged vote – incumbent vote share before floods, Lagged turnout – turnout in election before floods

Ψ p<0.1; * p<0.05; ** p<0.01; *** <0.001

**Flood impact on 2015 parliamentary election**

For the 2015 parliamentary election held in November, we used data on floods that took place in 2014 during February and May, and/or 2015 during May and October, which was in concordance with the time-frame used in previous research 36^,^^41^^,^^61^. Results showing the impact of the 2014-2015 floods on the 2015 parliamentary election can be seen in Table 2.

**Table 2:** Table 2. Flood effects on 2015 parliamentary election for matched and unmatched samples

	Unmatched data		Matched data	
	Non-flooded	Flooded	Non-flooded	Flooded
Unemployment	17.22 (8.69)	23.59*** (9.1)	23.03 (8.91)	23.59 (9.1)
Income	22505.18 (6155.99)	19270.14*** (5247.34)	19327.11 (5363.62)	19270.14 (5247.34)
Municipal income	2236.21 (1831.04)	1316.95*** (1011.99)	1135.46 (636.05)	1316.95^Ψ^ (1011.99)
Education	70.57 (10.94)	63.44*** (9.68)	64.09 (10.13)	63.44 (9.68)
Lagged turnout (2011)	59.04 (7.23)	59.76 (6.19)	59.36 (7.46)	59.76 (6.19)
Lagged vote (2011)	38.04 (15.29)	35.15* (10.87)	34.68 (15.60)	35.15 (10.87)
Turnout change (2015-2011)	-0.12 (6.30)	-1.53** (4.54)	-0.31 (6.69)	-1.53^Ψ^ (4.54)
Vote change (2015-2011)	-7.13 (11.85)	-5.34* (6.49)	-5.22 (8.39)	-5.34 (6.49)
N	402	154	154	154

Sample means with standard deviation in the parenthesis for: Unemployment – average percentage of unemployed individuals 2010-2012, Income – average yearly income per capita 2010-2012 (in HRK), Municipal income – average yearly municipal income per capita 2010-2012 (in HRK), Education – percentage of population aged 16-65 with high school of higher 2011, Lagged vote – incumbent vote share before floods, Lagged turnout – turnout in election before floods

Ψ p<0.1; * p<0.05; ** p<0.01; *** <0.001

Similar to the case of the presidential election, there was a significant difference in vote change before matching, but in the opposite direction – non-flooded areas showed a higher drop in incumbent vote share in the election following the floods. Also, the fall in turnout was more severe for the flooded areas. In addition, non-flooded areas had higher lagged turnout, lower unemployment rates, higher personal and municipal income, higher education levels, and higher lagged incumbent vote. Therefore, we repeated the matching procedure, which showed that all differences between the two subsamples disappeared after matching. Subsamples did not differ in any of the socioeconomic variables, lagged incumbent vote, nor vote change; the difference in turnout was marginally insignificant. To conclude, results indicated that floods did not appear to influence voting behaviour in the 2015 parliamentary election.

**Relief spending impact on 2015 parliamentary election**

Relief spending for floods in 2014 and 2015 was given during 2015, after the presidential and before the parliamentary election. Thus, data on government relief was used to investigate whether it had an impact on voting in the 2015 parliamentary election. Relief spending was approved for 103 out of 154 municipalities, and the amount of relief spending had a median of 3.1% of the amount of estimated flood damage. First, we investigated whether relief approval (approved vs. not approved) had an impact on vote change between the two elections by conducting a regression analysis with relief spending and other political and socio-demographic variables as predictors. The results are summarised in Table 3.

**Table 3:** Table 3. Predicting the parliamentary election vote change (2015-2011) based on the approval of relief spending, political and socio-demographic variables

	B	β
Intercept	-7.951 (5.156)	/
Lagged vote (2011)	-0.209 (0.057)	-0.348***
Relief approved	0.176 (1.409)	0.013
Income	-0.0002 (0.0001)	-0.128
Municipal income	-0.0009 (0.0005)	-0.136
Education	0.221 (0.079)	0.329**
Unenmployment	0.001 (0.079)	0.002
R^2^	0.183	/
N	154	/

Standard errors are in parenthesis; Lagged vote – incumbent vote share before floods, Relief approved – 0 (not approved) and 1 (approved), Income – average yearly income per capita 2010-2012 (in HRK), Municipal income – average yearly municipal income per capita 2010-2012 (in HRK), Education – percentage of population aged 16-65 with high school of higher 2011, Unemployment – average percentage of unemployed individuals 2010-2012

** p<0.01; *** <0.001

Regression model was statistically significant (F(6, 147)=5.494; p<0.001). Specifically, we could predict the change in vote for the incumbent (2015-2011) for the flooded areas based on the lagged incumbent vote (2011) and the educational structure of the local population. Keeping other variables constant, one percent vote increase for the incumbent in 2011 resulted in 0.21 percent fall in the 2015-2011 vote change; one percent increase in share of people with high school or higher education resulted in 0.22 percent increase in 2015-2011 vote change. However, relief approval did not predict vote change. Furthermore, we conducted regression analysis in order to predict vote change with the percentage of approved relief by including only those municipalities that received flood relief. Besides the percentage of approved relief, predictors were political and socio-demographic variables. The model was not significant (F(6, 96)=2.079; p=0.061). Therefore, it could be concluded that the presence of relief spending did not have an impact on vote change.

## Discussion

The backdrop of studies which focus on the impact of politically irrelevant influences on citizens’ behaviour is the concept of the (retrospectively) rational voter, according to which voters use elections as a means of punishing or rewarding incumbents. If they do this rationally, then their role in the political system is justified; i.e. the representative model of democracy is justified 62. However, if citizens are prone to making mistakes, like irrationally blaming the government for things which are not under its control, citizens’ role in the political system is not justified. Thus, we turn to other democratic models, such as elite, or minimalist democracy 63. A newer take on this discussion flourished with Achen and Bartel’s study 27^,^^28^ in which they showed that citizens punished the American incumbent president for shark attacks. Several other studies showed that citizens’ political behaviour is influenced by weather events, such as natural disasters 31^,^^32^^,^^33^^,^^35^ . They paint a picture of irrational citizens and point to the inability of elections to be adequate mechanisms of democratic accountability.

Following these studies, we attempted to replicate their findings for the case of floods in Croatia in 2014-2015. The goal of this research was to investigate whether floods, a form of natural disaster, had an impact on the following elections. Moreover, we wanted to check whether the government’s response to the floods, in the form of relief spending, had an impact.

It is important to note the methodological differences between present and several previous studies. In most of the previous studies, authors conducted regression analysis with the dependent variable usually being the change in incumbent vote share, the independent variable being the presence of a natural disaster, and several control variables, such as county size or lagged vote share 27^,^^32^^,^^33^^,^^35^^,^^64^. However, some authors have argued that this approach is not adequate because of the assumption that natural disasters function as random, exogenous shocks to the political system 42. Bodet, Thomas and Tessier 38 (p. 92) state clearly that “assuming the natural disaster acts as an as-if random exogenous shock, and treating it as a natural experiment, can lead to incorrect conclusions…studies that wish to treat natural disasters as experiments should justify this analytical choice by demonstrating the disaster produces equivalent experimental and control groups. Failing to do so would, in this case, lead us to inappropriately reject the null hypothesis”. Thus, it is possible to pinpoint the influence of natural disasters on voting behaviour only if flooded and non-flooded areas are comparable on variables that could have influenced the incumbent vote share. Moving from conceptual level toward the statistical one, the same problem arises when regression models are used in observational studies; i.e. “when there are large differences in distribution of covariates between treatment groups, adjusting for these differences with conventional multivariable techniques may not adequately balance the groups, and the remaining bias may limit valid causal inference” 52 (p. 954). One way to deal with this issue is to use matching method technique, which has been used in several previous studies 38^,^^42^^,^^55^ .

In the present study, when we compared the flooded and non-flooded areas, it seemed that the people from flooded areas punished the incumbents in both elections. However, once we accounted for differences in flooded and non-flooded areas in several sociodemographic and political variables, the effect disappeared. In other words, by using appropriate methodology (matching method technique), we found no effect of floods either on incumbent vote share or in the turnout for the following elections.

The conclusion following these results can fall into one of three categories. First, there is a possibility that floods usually have an impact on voting behaviour, but the current research has methodological issues, or the floods were not disastrous enough to elicit a response among the population of flooded areas. Considering the methodological issues, we feel that we have followed the above-mentioned suggestions on both the conceptual and statistical level. Also, we have obtained data from official sources considering vote shares, flood presence, and damage, as well as municipal level data on income, education, and unemployment. Considering that the floods studied in this paper were the biggest in (recorded) Croatian history, that they resulted in the destruction of several villages, and displacement of more than 13,000 people (and two dead)^65^ , we can conclude that they were disastrous enough to elicit a response. Even though it’s not unusual for studies to focus on a single case of a natural disaster (e.g. 36^,^^39^^,^^40^^,^^42^^,^^61^^,^^38^ ), the certainty of our conclusion would be improved by using a wider timeframe (e.g. 32^,^^35^^,^^41^).

Following the second argument from this category, that the effect of natural disasters was not found because the disaster was not big enough, was deemed an “act of God”, or the government was not blamed, etc. 27 , we think that this idea opens the possibility that we deem all null-results as being the result of a “bad” natural disaster, or to put it in other words – if the disaster would have been more extreme, we would have found an impact (for a similar discussion see 43 ).

A second possible conclusion drawn from the present results is related to the idea that natural disasters usually have an impact on political behaviour, but the negative response was counteracted by other variables, mainly concerning the government’s response to the disaster. Several studies have shown that natural disasters have a negative impact on the incumbent’s vote share in the following election 31^,^^32^^,^^33^^,^^39^ . Other studies have found that this negative impact can be lessened or counteracted by announcing a formal disaster declaration 31 , relief spending 32 , or by a general positive evaluation of the government’s performance following a disaster 39^,^^40^.

Moving back to our results; when we tried to predict voting behaviour within flooded areas, we found that the presence of relief spending had no effect on incumbent vote share. We could conclude that the relief was too low, or given too late, to counteract the impact of floods on the vote, but then we would expect to find the difference between flooded and non-flooded areas in vote change. There are several other possibilities. First, it is possible that some other aspect of voting behaviour was responsible for these results, such as the (dis)satisfaction with the way the government handled the floods. These individual-level variables were not available for this research, but even if they were, we would still then expect to see the overall effect of floods on incumbent vote share. Second, it is possible that we stumbled upon a floor effect. The incumbent party in the period 2011-2015 was the Social Democratic Party of Croatia (SDP) with its coalition partners. In 2011 SDP won 52.98% of votes, but only 37.09% in 2015. Thus, it is possible that the overall fall in support masked the effects of the floods. However, when we look at the presidential election, the incumbent in the period 2009-2014 was Ivo Josipović, who won 60.26% in 2009 and 49.26% in 2014. This difference in vote change is smaller for this election, and the overall incumbent vote share in 2014 is relatively high compared to the parliamentary election. Still, the flood effect on voting behaviour was absent in both elections. However, it’s important to note that the number of flooded areas was too small to use a matching method technique, so we used a regression analysis in which we tried to predict the incumbent vote change based on relief spending, several socio-demographic and political control variables. A suggestion for future studies would be to find cases of natural disasters within which there are enough cases of flooded areas that received and those that did not receive relief spending.

Finally, it is possible that floods indeed do not have an impact on the voting behaviour of citizens, and it seems our results point to this conclusion. However, we must attend to several issues. First, there are more studies published that found an effect of floods than vice versa 37^,^^43^^,^^67^ . This could be the case of publication bias 68 , or, as Fowler and Hall 43 pointed out and showed in their analysis, published articles could be based on false positive results. If that was the case, then one could expect that using more adequate methodology and statistical analysis would yield clearer results. Unfortunately, studies which have used matched methods have found negative flood effects on the incumbent vote 55 , positive effects 42 , and no effects 38 , as in the present study. Second, even though the goal of the study was to replicate previous findings in the previously unstudied context of floods in Croatia, we used two cases of natural disasters within a single country, which constrains the generalisation of our findings.

Thus, it seems that the verdict on the impact of natural disasters is not clear cut, and should not be taken as such. Even if we look at research done with adequate methodology, we find results going in all directions, with our results showing that floods did not have an impact on the incumbent’s vote share or voter turnout in two elections. Further research is needed, as well as opening up to publishing null results. First, we would suggest matched method as the methodology of choice for this type of research. Second, since results seem to vary in direction, it is probable that some moderator or mediator variables should be considered. For example, one could use type of natural disaster (which could be differently portrayed as “an act of God” versus “an act of Man” 69 , the incumbent’s approval in the period before the disaster, different legislature on dealing with and helping after natural disasters, public support for the affected areas, etc. Third, it could be useful to use more individual-level data, as in some previous studies. For example, survey studies can assess the way voters perceive the amount of destruction, as well as the way the incumbent government dealt with the disaster 39^,^^40^ . Studies can also combine individual level data with macro variables 36. Finally, we suggest using multilevel analysis, which can be used to combine data on political knowledge, political cynicism, ideology, etc. with county-level data to get a clearer idea of the impact of natural disasters on political behaviour.

## Conclusion

The goal of the present study was to study the impact of natural disasters on voting behaviour for the context of the 2014 and 2015 floods in Croatia. Results showed that, once socio-demographic and political differences between the flooded and non-flooded areas were controlled, floods did not have an impact on voting behaviour in the 2014 presidential or in the 2015 parliamentary election in Croatia. Furthermore, results indicated that neither presence of relief spending nor its amount had an impact on voting behaviour. However, keeping in mind the constraints of this study, further research is needed to get a clearer picture of the way natural disasters are related to political behaviour.

## Competing interests

The authors have declared that no competing interests exist.

## Corresponding author

Kosta Bovan: kosta.bovan@fpzg.hr

## Data Availability

All supplemental materials used for this article (data and R scripts) can be download via https://figshare.com/articles/Supplemental_materials_-_Do_natural_disasters_affect_voting_behavior_Evidence_from_Croatian_floods_/5821674

## References

[1] Bartels LM. Uninformed Votes: Information Effects in Presidential Elections. American Journal of Political Science 1996; 40(1): 194-230.

[2] Taber CS, Lodge M. Motivated Skepticism in the Evaluation of Political Beliefs. American Journal of Political Science 2006; 50(3): 755-769.

[3] Delli Carpini M, Keeter S. Stability and Change in the U.S. Public's Knowledge of Politics. Public Opinion Quarterly 1991; 55(4): 583-612.

[4] Zaller JR. The Nature and Origins of Mass Opinion. Cambridge: Cambridge University Press; 1992.

[5] Galston WA. Political Knowledge, Political Engagement, and Civic Education. Annual Review of Political Science 2001; 4: 217-234.

[6] Macedo S, Alex-Assensoh Y, Berry JM, Brintnall M, Campbell DE, Fraga LR, Fung A, Galston WA, Karpowitz CF, Levi M, Levinson M, Lipsitz K, Niemi RG, Putnam RD, Rahn WM, Reich R, Rodgers RR, Swanstorm T, Walsh KC. Democracy at Risk. How Political Choices Undermine Citizen Participation and What We Can Do About It. Washington: Brookings Institution Press; 2005.

[7] Jost JT, Sidanius, J. Political Psychology: An Introduction. In Jost JT, Sidanius, J, editors. Political Psychology. Key Readings. New York: Psychology Press; 2004. pp. 1-22.

[8] Ferejohn J. Incumbent performance and electoral control. Public choice 1986; 50(1–3): 5-25.

[9] Powell GB. Elections as Instruments of Democracy. Majoritarian and Proportional Visions. New Haven: Yale University Press; 2000.

[10] Anderson CJ. Economic voting and political context: a comparative perspective, Electoral Studies 2000; 19:151–170.

[11] Lewis-Beck MS, Stegmaier M. Economic determinants of electoral outcomes. Annual Review of Political Science 2000; 3(1): 183–219.

[12] Rapeli L. Who to punish? Retrospective voting and knowledge of government composition in a multiparty system. International Political Science Review 2016; 37(4): 407–421.

[13] Stevenson RT, Duch R. The meaning and use of subjective perceptions in studies of economic voting. Electoral Studies 2013; 32(2): 305–320.

[14] Ansolabehere S, Meredith M, Snowberg E. Mecro-Economic Voting: Local Information and Micro-Perceptions of the Macro-Economy. Economics & Politics 2014; 26(3): 380–410.

[15] Berry CR, Howell WG. Accountability and local elections: Rethinking retrospective voting. Journal of Politics 2007; 69(3): 844–858.

[16] Powell GB, Whitten GD. A Cross-National Analysis of Economic Voting: Taking Account of the Political Context. American Journal of Political Science 1993; 37(2): 391-414.

[17] Healy A, Malhotra N. Retrospective Voting Reconsidered. Annual Review of Political Science 2013; 16(1): 285–306.

[18] Davis OA, Hinich MJ, Ordeshook PC. An Expository Development of a Mathematical Model of the Electoral Process. American Political Science Review 1970; 64(2): 426–448.

[19] Rabinowitz G, Macdonald SE. A Directional Theory of Issue Voting. The American Political Science Review 1989; 83(1): 93-121.

[20] Westholm A. Distance versus Direction: The Illusory Defeat of the Proximity Theory of Electoral Choice. American Political Science Review 1997; 91(4): 865-883.

[21] Banai B, Pavela Banai I, Bovan K. Candidates’ voice in political debates and the outcome of presidential elections. Book of selected proceedings of XX. Psychology Days in Zadar; 2017; forthcoming.

[22] Pavela Banai I, Banai B, Bovan K. Vocal characteristics of presidential candidates predict the outcome of actual elections. Evolution and Human Behavior 2017; 38: 309-314.

[23] Gregory SW, Gallagher TJ. Spectral Analysis of Candidates’ Nonverbal Vocal Communication: Predicting U. S. Presidential Election Outcomes. Social Psychology Quarterly 2002; 65(3): 298-308.

[24] Rosenberg SW, Kahn S, Tran T. Creating a Political Image: Shaping Appearance and Manipulating the Vote. Political Behavior 1991; 13(4): 345-367.

[25] Surawski MK, Ossof EP. The effects of physical and vocal attractiveness on impression formation of politicians. Current Psychology 2006; 25(1): 15-27.

[26] Todorov A, Mandisodza AN, Goren A, Hall CC. Inferences of Competence from Faces Predict Election Outcomes. Science 2005; 308: 1623-1626. 10.1126/science.1110589 15947187

[27] Achen CH, Bartels LM. Blind retrospection: Electoral responses to drought, flu, and shark attacks. 2004. Available from: http://www.ethz.ch/content/dam/ethz/special-interest/gess/cis/international-relations-dam/Teaching/pwgrundlagenopenaccess/Weitere/AchenBartels.pdf

[28] Achen CH, Bartels LM. Democracy for realists: why elections do not produce responsive government. Princeton: Princeton University Press; 2016.

[29] Achen CH, Bartels LM. Blind retrospection: Why shark attacks are bad for democracy. 2012. Available from: https://my.vanderbilt.edu/larrybartels/files/2011/12/CSDI_WP_05-2013.pdf (Accessed: 27 March 2017).

[30] Healy AJ, Malhotra N, Mo CH. Irrelevant events affect voters’ evaluations of government performance. Proceedings of the National Academy of Sciences 2010; 107(29): 12804–12809. 10.1073/pnas.1007420107 PMC2919954 20615955

[31] Healy A, Malhotra N. Random Events, Economic Losses, and Retrospective Voting: Implications for Democratic Competence. Quartertly Journal of Political Science 2010; 5(2): 193–208.

[32] Gasper JT, Reeves A. Make It Rain? Retrospection and the Attentive Electorate in the Context of Natural Disasters. American Journal of Political Science 2011; 55(2): 340–355.

[33] Cole S, Healy A, Werker E. Do voters demand responsive governments? Evidence from Indian disaster relief. Journal of Development Economics 2012; 97(2): 167–181.

[34] Choudhury ZA. Politics of natural disaster: how governments maintain legitimacy in the wake of major disasters, 1990-2010. 2013. Available from: http://ir.uiowa.edu/etd/1566/.

[35] Chang CP, Berdiev AN. Do natural disasters increase the likelihood that a government is replaced?. Applied Economics 2015; 47(17): 1788–1808.

[36] Sinclair B, Hall TE, Alvarez RM. Flooding the vote: Hurricane Katrina and voter participation in New Orleans. American politics research 2011; 39(5): 921–957.

[37] Fair C et al. Economic Shocks and Civic Engagement: Evidence from the 2010-11 Pakistani Floods. 2014. Available from: http://citeseerx.ist.psu.edu/viewdoc/download?doi=10.1.1.640.735&rep=rep1&type=pdf

[38] Bodet MA, Thomas M, Tessier C. Come hell or high water: An investigation of the effects of a natural disaster on a local election. Electoral Studies 2016; 43: 85–94.

[39] Arceneaux K, Stein RM. Who is Held Responsible When Disaster Strikes? The Attribution of Responsibility for a Natural Disaster in an Urban Election. Journal of Urban Affairs 2006; 28(1): 43–53.

[40] Lay JC. Race, Retrospective Voting, and Disasters: The Re-Election of C. Ray Nagin after Hurricane Katrina. Urban Affairs Review 2009; 44(5): 645–662.

[41] Healy A, Malhotra N. Myopic Voters and Natural Disaster Policy. American Political Science Review 2009; 103(03): 387–406.

[42] Velez Y, Martin D. Sandy the Rainmaker: The Electoral Impact of a Super Storm. PS: Political Science & Politics 2013; 46(2): 313–323.

[43] Fowler A, Hall AB. Do Shark Attacks Influence Presidential Elections? Reassessing a Prominent Finding on Voter Competence. 2016. Available from: https://polmeth.wustl.edu/files/polmeth/fowlerhall_sharks.pdf

[44] Fowler A, Montagnes BP. College football, elections, and false-positive results in observational research. Proceedings of the National Academy of Sciences 2015, 112(45): 13800–13804. 10.1073/pnas.1502615112 PMC4653178 26504202

[45] Novosel, T. Obrana od poplava na slivu rijeka Kupe i Save [Flood defense at rivers Kupa and Sava]. Hrvatska vodoprivreda 2014; 206: 16-21.

[46] Fortuna Višnecki, N. Pomoć i potpora Oružanih snaga RH poplavnom području [Croatian Armed Forces help and support flooded areas]'. Hrvatska vodoprivreda 2014; 207: 51-53.

[47] Plišić, I. Proslov Generalnog direktora Hrvatskih voda [Foreword of Croatian waters' General director]. Hrvatska vodoprivreda 2014; 207: 4-5.

[48] Hrvatska, zemlja velikog srca - poruka stradalima: Niste sami! [Croatia, a big-hearted country - message to stricken: You are not alone!]. (2014, May, 27). Retreived from http://hrtprikazuje.hrt.hr/245767/hrvatska-zemlja-velikog-srca-poruka-stradalima-niste-sami

[49] Hrvatske vode najveći su krivac, nije bilo dosta vreća i pijeska [Croatian Waters are the main culprit, there were not enough bags and sand]. (2014, May 19). Retreived from http://www.vecernji.hr/hrvatska/hrvatske-vode-najveci-su-krivac-nije-bilo-dosta-vreca-pijeska-i-sljunka-939646

[50] Novosel, T. Obrana od poplava u 2015. godini [Flood defense in 2015]. Hrvatska vodoprivreda 2015; 213: 5-11.

[51] Austin PC, Mamdani MM. A comparison of propensity score methods: a case-study estimating the effectiveness of post-AMI statin use. Statistics in Medicine 2006; 25(12): 2084–2106. 10.1002/sim.2328 16220490

[52] Newgard CD et al. Advanced Statistics: The Propensity Score-A Method for Estimating Treatment Effect in Observational Research’. Academic Emergency Medicine 2004; 11(9): 953–961. 10.1197/j.aem.2004.02.530 15347546

[53] Rubin DB. Using propensity scores to help design observational studies: application to the tobacco litigation’, Health Services and Outcomes Research Methodology 2001; 2(3): 169–188.

[54] Caliendo M, Kopeinig S. Some Practical Guidance for the Implementation of Propensity Score Matching’. Journal of Economic Surveys 2008; 22(1): 31–72.

[55] Heersink B, Peterson BD, Jenkins JA. Disasters and Elections: Estimating the Net Effect of Damage and Relief’ 2016. Available at: http://www.borisheersink.com/s/Disasters-and-Elections-v6.pdf (Accessed: 27 March 2017).

[56] Baser O. Too Much Ado about Propensity Score Models? Comparing Methods of Propensity Score Matching. Value in Health 2006; 9(6): 377–385. 10.1111/j.1524-4733.2006.00130.x 17076868

[57] Ho DE, Imai K, King G, Stuart EA. MatchIt: Nonparametric Preprocessing for Parametric Causal Inference. Journal of Statistical Software 2011; 42(8): 1-28.

[58] Wickham H, Francois R. dplyr: A Grammar of Data Manipulation. R package version 0.5.0. 2016 https://CRAN.R-project.org/package=dplyr

[59] Behrendt S. lm.beta: Add Standardized Regression Coefficients to lm-Objects. R package version 1.5-1. 2014. https://CRAN.R-project.org/package=lm.beta

[60] Fox J, Weisberg S. An R Companion to Applied Regression 2011. Thousand Oaks CA: Sage.

[61] Bechtel MM, Hainmueller J. How Lasting Is Voter Gratitude? An Analysis of the Short- and Long-Term Electoral Returns to Beneficial Policy’. American Journal of Political Science 2011; 55(4): 852–868.

[62] Mill JS. Considerations on Representative Government. London: The Electric Book Company; 1861|2001.

[63] Schumpeter JA. Capitalism, Socialism and Democracy. New York; Florence: Routledge Taylor & Francis Group; 1943.

[64] Potluka O, Slavikova L. Impact of floods on local political representation. Acta Politologica 2010; 2(1): 1–17.

[65] Poplave u Vukovarsko-srijemskoj županiji u svibnju 2014 [May 2014 floods in Vukovar-Srijem county]. 2015, January 16. Retreived from: http://www.duzs.hr/news.aspx?newsID=22140&pageID=677

[66] Poplave u Vukovarsko-srijemskoj županiji u svibnju 2014 [May 2014 floods in Vukovar-Srijem county]. 2015, January 16. Retreived from: http://www.duzs.hr/news.aspx?newsID=22140&pageID=677

[67] Abney FG, Hill LB. Natural disasters as a political variable: The effect of a hurricane on an urban election. American Political Science Review 1966; 60(4): 974-81.

[68] Franco A, Malhotra N, Simonovits G. Publication Bias in the Social Sciences: Unlocking the File Drawer. Science 2014; 345(6203): 1502–1505. 10.1126/science.1255484 25170047

[69] Dodds GG. “This Was No Act of God:” Disaster, Causality, and Politics. Risk, Hazards & Crisis in Public Policy 2015; 6(1): 44–68.

[70] Act on Protection against Natural Disasters, 73/97 N.N. 1997; Retrieved from: https://www.zakon.hr/z/540/Zakon-o-za%C5%A1titi-od-elementarnih-nepogoda

[71] Methodology for making threat assessments, N.N. 96/98; 1998. Retrieved from: http://narodne-novine.nn.hr/clanci/sluzbeni/1998_07_96_1330.html

